# Supplementary material for: Small Polar Molecules: A Challenge in Marine Chemical Ecology
Source: Molecules. 2018 Dec 31;24(1):135. doi: 10.3390/molecules24010135 (PMC6337545; doi:10.3390/molecules24010135)
Supplement: Supplementary file 1 [file molecules-24-00135-s001.pdf]

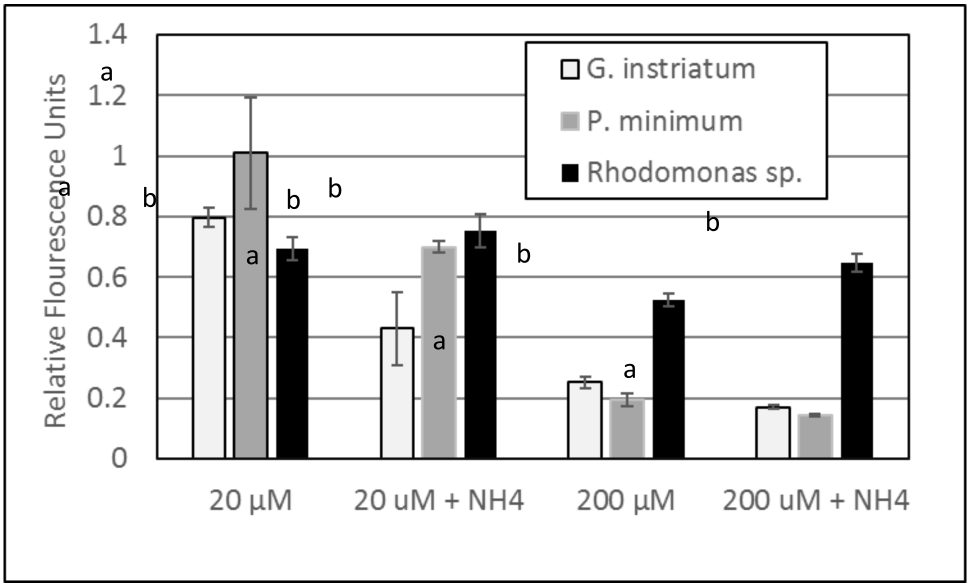

Figure 1. Bar graph showing the relative fluorescence of two dinoflagellates and a cryptophyte in response to *n*-butylamine alone 20 and 200 µM or to synergistic effects of n-butylamine and ammonium (100 µM). The letters a, b and c indicate significant differences ($p < 0.05$).
